# Computer-Assisted Navigation in Reverse Shoulder Arthroplasty: Surgical Experience and Clinical Outcomes

**DOI:** 10.3390/jcm13092512

**Published:** 2024-04-25

**Authors:** Luca Andriollo, Silvia Pietramala, Alberto Polizzi, Giuseppe Niccoli, Guido Zattoni, Vincenzo Morea

**Affiliations:** 1 Orthopedics and Traumatology, Fondazione Poliambulanza Hospital, 25124 Brescia, Italy; 2 Department of Orthopedics, Catholic University of the Sacred Heart, 00168 Rome, Italy

**Keywords:** computer-assisted navigation, reverse shoulder arthroplasty, GPS navigation, shoulder replacement, shoulder osteoarthritis

## Abstract

**Background:** The primary cause of medium- to long-term complications in reverse shoulder arthroplasty (RSA) is the failure of the glenoid component. The purpose of this study was to evaluate both the achievement of planning through computer-assisted navigation and the clinical outcomes at a minimum follow-up (FU) of 12 months. **Methods:** From December 2019 to December 2022, 57 Equinoxe RSAs with computer-assisted navigation were performed. The average age was 72.8 ± 6.6 years. Using the Orthoblue software, the version and inclination of the glenoid were evaluated from a preoperative CT scan, and planning was performed. Intraoperative navigation data were evaluated, and the clinical outcomes were assessed at a minimum follow-up of 12 months. **Results:** The average follow-up was 30.7 ± 13.5 months. The planning was reproduced in all implants. No errors in the computer-assisted navigation system were detected. No intraoperative or postoperative complications were recorded. At the final FU, the average active anterior elevation was 143° ± 36°, external rotation was 34° ± 5°, QuickDASH score was 19 ± 16 points, and constant score was 77 ± 18. **Conclusions:** Computer-assisted navigation is a reliable system for positioning prosthetic implants on challenging glenoids. A longer follow-up period is necessary to confirm the reduction in postoperative complications and the increase in survival compared to traditional RSA.

## 1. Introduction

With the increasing indications and frequency of reverse shoulder arthroplasty (RSA), it becomes crucial to reduce both intraoperative and postoperative complications [1,2]. 

Despite being a relatively recent technology, intraoperative computer-assisted navigation is becoming more and more acknowledged among surgeons. This technique enables surgeons to precisely position instruments and implants in alignment by starting with a preoperative plan, respecting the anatomy and the unique characteristics of the patient [3,4].

Positioning the glenoid component can pose challenges, including limited exposure, difficulty visualizing the deep regions of the glenoid vault, and variations in glenoid morphology like retroversion and bone deficiency [5,6].

It is estimated that, every year, almost 1% of implanted shoulder prostheses end up in surgical revision for aseptic loosening, which is mainly related to the malposition of the glenoid component [7,8].

Studies have demonstrated that preoperative templating software alone enhances the precision of implant placement. Unlike patient-specific guides, navigation does not necessitate custom manufacturing and is readily available post-preoperative planning [2,7]. 

Augmentation of the baseplate, along with different degrees of version and fewer screws, is a factor related to better clinical outcomes, but often, surgeons are afraid to proceed in this way [9,10]. The diffusion of intraoperative navigation is related to an increased use of augmentation of the baseplate and fewer screws, which leads to better clinical outcomes and decreased revision rates. 

Despite its ability to accurately execute a preoperative plan, the clinical advantages of intraoperative navigation, including improved outcomes, reduced complications related to glenoid misalignment, and long-term implant durability, are yet to be fully understood. 

Recently, some studies have been published in the literature trying to determine the superiority of computer navigation in RSA, but the topic is still debated [9,11].

The aim of our study is to provide further examples of how computer-assisted navigation in RSA is reliable, lacking in significant complications, and a useful tool to improve precision and clinical outcomes, even with expert surgeons.

## 2. Materials and Methods

Patients treated using Equinoxe RSA (Exactech, Gainesville, FL, USA) with computer-assisted navigation between December 2019 and December 2022 were retrospectively evaluated. All procedures were performed at a single center by two surgeons experienced in shoulder arthroplasties.

The inclusion criteria were as follows: severe glenoid deformity due to osteoarthritis with B2-B3 or C-type glenoid according to Walch, irreducible inveterate dislocation with glenohumeral deformity, and multi-fragmentary proximal humerus fracture in the presence of symptomatic glenohumeral osteoarthritis [12]. Exclusion criteria included pathological fractures, a follow-up of less than 12 months, and a loss of follow-up data. The preoperative diagnosis required the execution of a standard antero-posterior shoulder X-ray (axial and outlet view). Additionally, a preoperative CT scan was performed on all patients, which was necessary for the use of the navigation system.

In all patients, an Equinoxe Reverse System prosthesis (Exactech, Gainesville, FL, USA) was implanted following preoperative planning with Orthoblue software—Equinoxe Shoulder Planning App (Exactech, Gainesville, FL, USA) and intraoperative application of the planning through the computer-assisted navigation system ExactechGPS Shoulder Application (Exactech, Gainesville, FL, USA). Accuracy was defined as a reproduction of the planning within ±1°.

Demographic and preoperative data were collected, including the planned inclination and version. Additionally, data concerning in-hospital complications, length of hospital stay, hemoglobin levels, any transfusions, surgical time, actual and validated inclination and version, type of glenoid baseplate and glenosphere used, as well as the quantity and length of the screws utilized, were collected.

The assessment also included the evaluation of acute complications, such as post-surgical local hematoma, vascular injury, or nerve injury, as well as follow-up complications, including readmission or reoperation rates and their respective causes, such as infection, dislocation, aseptic mobilization, and instability.

Preoperatively and at the final follow-up, all patients underwent a clinical examination that included patient-reported outcome measures (PROMs) such as the QuickDASH score and constant score, in addition to the Visual Analogue Scale (VAS). Degrees of range of motion (ROM) in anterior elevation, abduction, and external rotation were also evaluated. The data from the functional outcomes preoperatively and at the final follow-up were compared.

At the final follow-up, radiographs were evaluated to assess the presence of areas of radiolucency around the prosthetic implant, both at the humeral and glenoid levels, and any potential mobilization of the implant itself.

Patients received routine venous thromboembolism prevention with low-molecular-weight heparin for 20 days. Alternatively, chronic anticoagulant therapy was administered as an option. Furthermore, Cefazolin was used as a routine perioperative prophylactic antibiotic. In detail, Cefazolin 2 g was administered intravenously 30 min before the surgical procedure, and Cefazolin 1 g was administered intravenously every 12 h for the 36 h following the surgery.

Postoperative rehabilitation protocols included immediate passive mobilization up to 90° in abduction and elevation, isometric deltoid exercises, full elbow flexion and extension, and no rotation. An adduction sling was used for the first 30 days. Rotational movements were gradually introduced after one month from the surgery.

### 2.1. Surgical Technique

#### 2.1.1. Preoperative Time

Exactech (Gainesville, FL, USA), using GPS navigation, has developed proprietary software to assist in preoperative planning for the Equinoxe Reverse System prosthesis. The system uses preoperative CT scans to generate a three-dimensional model of the native scapula.

**Figure 1:**
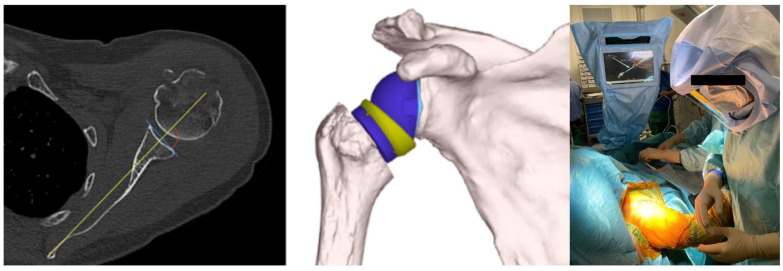
The surgical procedure for reverse shoulder arthroplasty with GPS navigation involves a preoperative CT scan, planning, and intraoperative navigation.

Using the Orthoblue software—Equinoxe Shoulder Planning App (Exactech, Gainesville, FL, USA), the glenoid component can then be virtually positioned within the 3D rendering of the patient’s scapula before surgery begins. It is easy to visualize the version, inclination, and positioning of the glenoid implant, evaluating the need for augmentations, increasing and compensating for the inclination and version. It is also possible to determine the measurement of the lateralization and elongation of the humeral stem in relation to the acromion and the native glenoid rotation center and compare them with the measurements corrected by the proposed implant. In addition, it is possible to evaluate the size of the glenosphere, the lateralization and the size of the insert, the range of motion, and, thus, the excursion of the humerus on the scapula in terms of adduction and abduction, internal and external rotation, anterior elevation, and retroposition of the proposed implant (Figure 1).

The system allows identifying the contact point between the humeral implant and the scapula and highlighting the critical point so as to modify the positioning of the components and make corrections to obtain maximum joint recovery.

The GPS technology uses a fixed point on the patient’s anatomy, in this case a tracker attached to the coracoid, combined with inputs provided by the surgeon through an electronic handpiece that identifies a bone map for the correct implantation of the components along the Friedmann axis.

The decision to implant a reverse arthroplasty is contingent upon the execution of thin-layer CT uploaded to the Orthoblue software (Exactech, Gainesville, FL, USA) for GPS reconstruction. The GPS data are made available to the surgeon in a short time from 3 to 7 days after acquisition.

The final position of the glenoid implant is guided by the GPS system only if requested through the involvement of Orthoblue and transferred via a USB module to the GPS hardware on the day of the surgery.

#### 2.1.2. Surgical Time

The patient is positioned on the operating table in a beach chair position. The computerized support with a touch screen monitor is included within the surgical field, placed by a special support, and protected by a sterile drape.

The surgical incision extends 2–3 cm proximally compared to the classic deltopectoral approach to allow the correct exposure of the coracoid. The humeral osteotomy and its preparation occur through the classic technique guided by preoperative planning. Subsequently, the procedure involves the isolation and preparation of the upper portion of the coracoid for the housing of the tracker. Two screws are used, with the first longer one placed at the base of the coracoid and the second shorter one at the apex. The exposure of the glenoid and the accurate removal of the soft tissues and remnants of the joint capsule, as required by the surgical technique, follow.

The next step is the matching of the preoperative 3D CT with the patient’s anatomical references, and this is performed using an electronic pen with a tracker to be carefully placed on the landmarks indicated by the software.

At the end of the acquisition process, the software analyzes the accuracy of the anatomical references: in case of poor accuracy, the procedure must be repeated, and this can be performed by choosing to repeat the entire procedure or only some less accurate steps detected at the end of the process. After registering the anatomical references, the operative phase begins, and the instrumentation allows for the control of the position of the pilot hole for the central peg, the depth of the glenoid reaming, and the direction of the drilling of the central peg and screws by also measuring the depth of the drill penetration and, therefore, the relative screw length.

The positioning of the definitive glenoid component and screws is performed with the help of GPS and allows for the evaluation of the orientation of the baseplate as planned and measuring the length of the screws. The Equinoxe Glenoid System has 6 holes for compression screws with an allowed excursion of 30° stabilized with an additional locking screw. In our experience, 2–3 screws have been used in all implants (1 in the upper hole and 2 or 3 in the lower ones). The direction of the drill for the positioning of the screws is controlled by GPS. The length of the screws is also checked through a color system present on the tip of the drill. Once this phase is completed, the coracoid tracker is removed, and the glenosphere is positioned using the standard technique.

**Figure 2:**
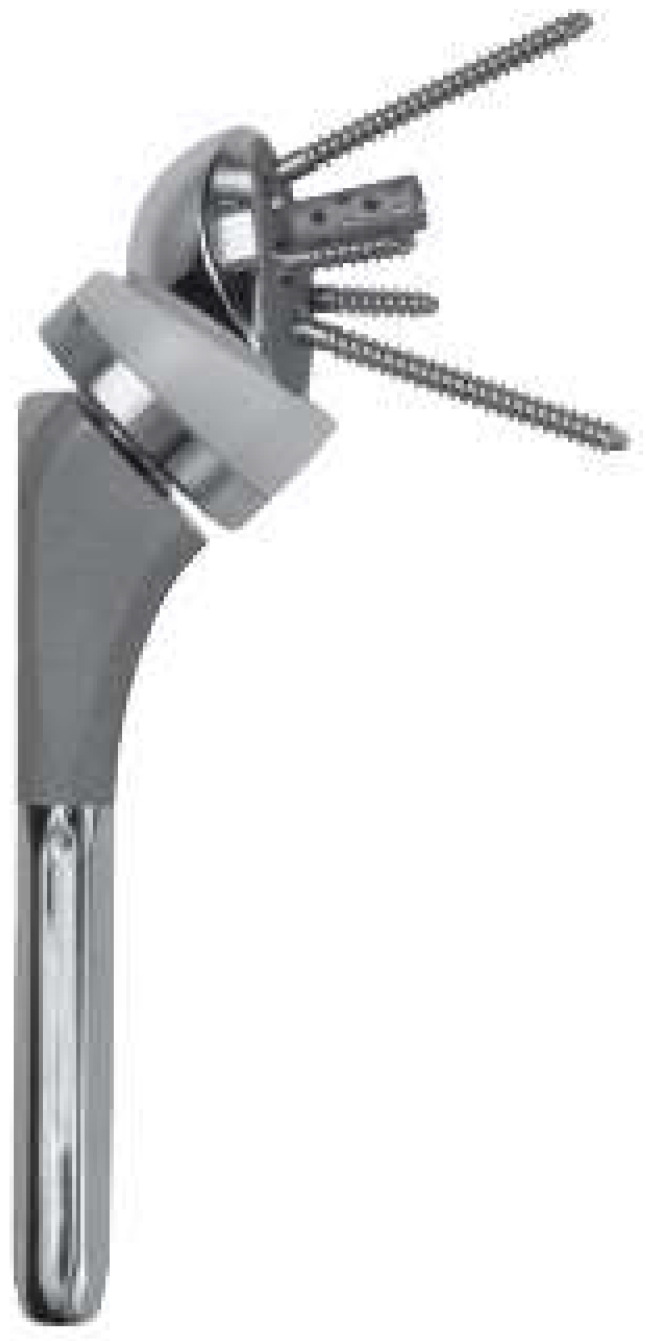
Equinoxe Reverse System (Exactech, Gainesville, FL, USA) implant.

The humeral component is positioned with traditional instrumentation, as is the reduction in the implant. Furthermore, it is possible to choose the size of the insert variable in length but also in containment; that is, it is possible to choose a more enveloping anti-dislocation-constrained insert to ensure more stability in implants at high risk of instability (Figure 2).

Although the use of GPS and traditional preoperative planning software based on CT provides excellent clinical results, it is necessary to complete the surgical technique with GPS assistance to the implant of the humeral component and estimate the tension of the components through the assistance of dedicated instrumentation.

### 2.2. Statistical Analysis

Statistical analysis was performed using SPSS v18.0 (Chicago, IL, USA) by an independent statistician. Continuous variables are reported using averages and standard deviations (SDs), while categorical variables are presented using frequency distributions and percentages. Biserial correlations were performed using a two-tailed test. *p* < 0.05 was considered a significant difference.

Level of evidence III: retrospective cohort study.

## 3. Results

From December 2019 to December 2022, 57 Equinoxe RSAs with computer-assisted navigation were performed. A retrospective assessment was conducted on a total of 56 patients, which included 22 males (39.3%) and 34 females (60.7%). The average age at the time of surgery was 72.8 ± 6.6 years. By the final follow-up, at 12 months, one patient (1.8%) had passed away due to causes unrelated to the surgery, and no patient was excluded due to a lack of data.

Ten cases (17.9%) involved the left shoulder, while forty-six (82.1%) involved the right shoulder. The reasons for treatment were glenohumeral osteoarthritis in 49 patients (87.5%), irreducible inveterate dislocation in 4 (7.1%), and proximal humerus fracture in 3 (5.4%). The baseline demographics are detailed in Table 1.

The average surgical time was 102 ± 16 min. The average lower inclination was 2.4° ± 2.8°, while the average retroversion was 2.2° ± 2.7°. With an accuracy of ±1°, the planning was reproduced in 100% of the cases.

With the goal of achieving proper joint stability through the attainment of adequate offset, in seven patients (12.5%), a humeral liner with 2.5 mm of offset was used, while in two patients (3.6%), a humeral liner with 4 mm of offset was utilized. The intraoperative data are reported in Table 2.

The preoperative hemoglobin levels averaged 13.1 ± 1.2 g/L, while the first-day postoperative hemoglobin was 11.4 ± 1 g/L and discharge hemoglobin was 10.6 ± 0.9 g/L. Packed red cells were transfused in two patients (3.6%).

The average duration of hospital stay was 3.7 ± 0.7 days. No in-hospital complications were observed.

The average follow-up duration was 30.7 ± 13.5 months. No complications were reported at follow-up, including readmission or reoperation rates and their respective causes, such as infection, dislocation, aseptic loosening, and instability. No cases of scapular notching or intraoperative fractures were reported.

**Figure 3:**
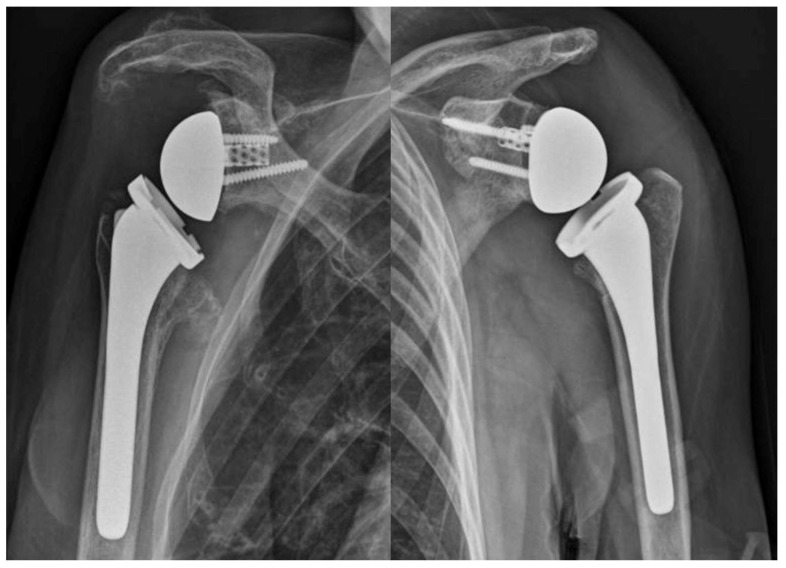
X-rays at the final follow-up.

At the final follow-up radiographic evaluation, no areas of radiolucency, peri-implant bone resorption, or device mobilization were detected (Figure 3).

At the final follow-up, the average active anterior elevation was 143° ± 36°, external rotation was 34° ± 5°, and anterior elevation was 143° ± 36°. The QuickDASH at the final follow-up was 19 ± 16 and the constant score was 77 ± 18, while the VAS was 0 ± 0.7. The data on ROM and PROMs at follow-up, compared with the preoperative data, showed a statistically significant improvement in all analyzed parameters. The data related to the outcomes are reported in Table 3.

## 4. Discussion

GPS navigation to reverse shoulder arthroplasty is gaining its place in clinical practice: this study serves as an example of applying GPS navigation to reverse shoulder arthroplasty, with a particular focus on how preoperative planning and intraoperative measurements correlate with more satisfactory clinical outcomes. The main purpose of this study was to assess this technique as reliable, easily reproducible, and precise to help both young and experienced surgeons offer patients better results in terms of function and return to daily life activities, validating the increasing interest in this technique.

The recent literature has seen studies advocating for GPS navigation in surgical procedures [11,13,14,15,16]. However, the body of literature on intraoperative navigation in shoulder prosthetics remains relatively limited due to its recent emergence as a topic of interest. As an area that has not been extensively explored, the existing literature has mainly concentrated on how GPS navigation could enhance specific aspects of surgical practice, but conclusive evidence regarding the definitive superiority of this technique over standard methods is lacking.

Our study centers on a patient cohort treated by a single surgical team, attributing greater significance to the GPS system in effecting actual improvements rather than solely relying on individual surgeon expertise. Indeed, navigation has been demonstrated to enhance the precision of glenoid component positioning and screw fixation, even with experienced surgeons.

This study represents one of the largest follow-up investigations, analyzing data on some of the main concerns in the literature towards GPS navigation: the increased surgical time, the complication rate related to the implant, and the learning curve.

As regards surgical time, our study pointed out a mean surgical time of 102 min, in line with what is reported in the literature for conventional RSA [15]. The length of hospital stay was 3.2 days on average, which is higher than that reported in the literature but coherent with the management of prosthesis hospitalization in our center [13,17]. 

As for implant-related complications, there is a slight chance of coracoid fracture related to the implant attached to the glenoid, as reported in the literature, especially in patients with severe osteoporosis or altered coracoid anatomy [18].

Among the other complications reported, there are glenoid loosening, dislocations, and unexplained pain. In our cohort, for the whole length of the follow-up, we reported no complications related to the implant. We are aware of the small size of the sample, but the data described in the literature confirm that, overall, this technique has a low complication rate (1.7% overall complication rate) [9,13,19].

Also, in cases where surgeons had considerable expertise, as the ones in our team did, GPS navigation was considered useful, especially in cases of complex glenoid anatomy (70% of cases were Walch B3 and C), in which this system already showed applicability [20].

It was stated how not only the implant size but also the overall position of the implant plays a crucial role in clinical outcomes.

In a recent multicentric study, attention was focused on the degree of version [13]. In particular, it was stated how a version of more than 15° is related to better clinical outcomes and, in particular, to active forward elevation, internal rotation, and different clinical stores. 

However, in our study, the mean version was 2.2° but we reported similar results of forward elevation, while the constant score was higher in our cohort. 

Regarding version, values under 10° are the most frequently observed. Younderian A.R et al., for instance, documented version of less than 10° in 80 out of 413 patients. While their findings suggesting that better outcomes are associated with higher degrees of version are intriguing, they require thorough analysis and validation with a larger patient cohort [13]. As for the constant score we recorded, the follow-up we recorded was shorter than the one reported in other papers that registered lower constant scores than ours [11,13]. As regards traditional RTSA, the average values of constant score reported by these two large multicenter studies were 63, 65 and 68 [13], and both these studies reported a 2-year follow-up. It must be said that the number of patients who underwent manual RTSA is larger in many comparative studies, so this should also be taken into account.

Baseplate augmentation is a useful tool to manage complex glenoid abnormalities, and recent studies have shown how an augmented baseplate is linked to better clinical outcomes [16]. The use of augmented glenoid implants has grown since preoperative planning began to be used, and also, in our study, augmentation was planned in 67.9% of cases. We did not investigate the correlation between augmentation and better outcomes as it was not an aim of our study, but we followed intraoperative planning in 100% of cases reporting statistically significant results in the clinical outcomes at follow-up.

In traditional RSA, surgeons often feel more confident to perform fixation with a larger number of screws, but this could affect bone stock, costs, and surgical time in a negative way. A navigation system requires the use of fewer screws, as also reported in our study [10]. In fact, in almost 90% of cases, only two screws were used. 

All cases of the intraoperative data we registered, concerning implant positioning, glenoid retroversion, and glenosphere lateralization, reproduced the initial preoperative plan with a positive resonance on clinical outcomes. 

This is especially important in RSA in which limitations in postoperative to internal and external rotation are described [21].

According to the literature, the improvement in external rotation after a traditional RSA is often limited and not significant [22]. In our cohort, we registered an increase in ER of more than 10° (*p* < 0.001) compared to the preoperative findings, and this result was also observed by Youderian et al. in a larger sample [13]. 

On the other hand, to our knowledge, this is the first study in which the postoperative anterior elevation increased in a significant way in computer-assisted navigation RSA.

One of the most significant limitations of this study is the short-term outcomes. Considering the observed medium follow-up, we can only assess positive results regarding intraoperative and immediate complications, like early dislocations and glenoid fractures as well as untreatable pain. A longer follow-up is needed to assess the absence of late-term complications such implant loosening and revision rates.

A point of strength of this study is that each surgery was performed by surgeons belonging to the same team and with the same level of experience, meaning that the results were not influenced by different techniques, implant choices, and knowledge of basic surgical techniques.

Comparing these results to traditional RSAs performed by the same surgeons on patients with the same characteristics could bring more value to the results we found.

Regarding the humeral stem, currently, only planning is allowed with the possibility to verify joint movement on various planes and the potential for impingement. From a future perspective, it is hypothesized that with the introduction of humeral navigation, there may be a further upgrade in the accuracy of implant positioning. Indeed, the goal of technological development is always to reduce long-term complications and improve the quality of functionality of RSA, with the most important aim being a positive impact on the patient’s quality of life.

This study presents an example of the reliability of computer-assisted RSA, in particular using Equinoxe computer-assisted RSA. The excellent results related to this technology led to the development of even more advanced techniques such as CT scan-based planning and robot-assisted surgery. As regards robot-assisted surgery, it is not possible to properly compare the two methods because of the lack of available, eligible, peer-reviewed studies with a high-quality level of evidence. A recent published case report supports the good expectations regarding this technology [23]. Moreover, as described in the literature, a new advancement in robotic-assisted TSA involves the implementation of “force-space navigation” using a Stewart Platform robot equipped with a distinctive motion-tracking system [24].

## 5. Conclusions

Computer-assisted navigation is a dependable system for positioning prosthetic implants on challenging glenoids. The medium-term results of clinical outcomes and survival have proven promising.

A longer follow-up is necessary to confirm the reduction in postoperative complications, the high levels of PROMs achieved, and the increase in survival compared to RSAs implanted with the traditional technique.

Further clinical outcomes are expected from the possibility of humeral stem navigation.

## Figures and Tables

**Table 1:** Demographic data.

**Patient Population**	**Number**	**%**
Total no.	57	100
Died	1	1.8
Available	56	98.2
**Sex**	**Number**	**%**
Male	22	39.3
Female	34	60.7
**Age**	**Average (Y)**	**SD**
	72.8	6.6
**Side**	**Number**	**%**
Left	10	17.9
Right	46	82.1
**Indication**	**Number**	**%**
Osteoarthritis	49	87.5
Walch B2	15	30.6
Walch B3	21	42.9
Walch C	13	26.5
Inveterate dislocation	4	7.1
Proximal humerus fracture	3	5.4

**Table 2:** Intraoperative data.

**Surgical time**	**Average (minutes)**	**SD**
	102	16
**Lower inclination**	**Average (degrees)**	**SD**
	2.4°	2.8°
**Retroversion**	**Average (degrees)**	**SD**
	2.2°	2.7°
**Baseplate**	**Number**	**%**
Standard	18	32.1
8° posterior augment	30	53.6
10° superior augment	8	14.3
**Glenosphere size**	**Number**	**%**
38 mm	51	91.1
42 mm	5	8.9
**Humeral liner offset**	**Number**	**%**
0 mm	47	83.9
2.5 mm	7	12.5
4 mm	2	3.6
**Number of screws**	**Number**	**%**
2	50	89.3
3	6	10.7
**Length of screws**	**Average (mm)**	**SD**
	32.9	5.7

**Table 3:** Outcomes at the final follow-up.

Outcomes	Preoperative	Final Follow-Up	*p* Value
Anterior elevation	85° ± 17.3°	143° ± 36°	<0.001
Abduction	89° ± 21.5°	146° ± 27°	<0.001
External rotation	24.7° ± 8.3°	35° ± 5°	<0.001
QuickDASH	76 ± 23	19 ± 16	<0.001
Constant score	37 ± 21	77 ± 18	<0.001
Visual Analogue Scale	6.8 ± 2.7	0 ± 0.7	<0.001

## Data Availability

The data presented in this study are available on request from the corresponding author (privacy).
